# Meat

**Published:** 1906-09

**Authors:** 


### MEAT.

THE effect of exposing the methods of the beef packers and the wave of nausea that swept over the country following it have been felt in Atlanta, and the city council, acting in conjunction with the Board of Health, has taken the matter in hand as affects the local situation and have passed resolutions which, if followed out, will insure for the city clean and wholesome meat. Those who have taken oath that they will eat no meat while the Lord liveth may safely relinquish their Nebuchadnezzar-like grazing and return to the flesh-pots of Egypt with every reasonable assurance that a bite into a piece of steak will not disclose a tuberculous sinus nor will the discussion of a mess of pork offer a practical experiment in the transplantation of trichinæ or tapeworm.

The great American stomach, the chief seat of the emotion, has been trifled with, flaunted and deeply offended. The stomach has a way when maltreated of discharging its contents, frequently without the slightest regard for conventionality. In the instance of the Beef Trust disclosures, we have a picture of fomential regurgitation with the packers floundering about in it with the feeble swimming stroke of a bluebottle in a bucket of swill. It is likely that with this submerging evidence of public disapproval, they will mend their ways and the citizens of the great republic will no longer unwittingly usurp the homely function of the buzzard and subsist on carrion.

---

THE Board of Health of the city of Atlanta has been doing efficient work in the extermination of mosquitoes, and already the public is beginning to notice the relatively small number of the winged pests that usually at this season of the year disturb slumber and disorder the tranquillity of front-porch rumination. The plan adopted consists in screening sewers and water-tanks and the free use of kerosene oil on the surface of dead water, together with admonition to householders to clear out mosquito generators. Disease-bearing mosquitoes exist sparingly in the city, and its vicinity, and it would appear to be no very difficult matter to suppress them altogether if the proper precautions are taken. Physicians should inform their patients of the importance of not allowing any water in an exposed situation to stand for any length of time. This applies to flower-pots and tubs, and such odments in the back premises as are capable of holding water and allowing an incubating medium for the female mosquito's accouchement. A kick on a battered coffeepot in the weeds may save many slaps and objurgatory remarks directed at the hungry insect. If the public will have these small details in mind and carry them out properly, the work of the health authorities will be greatly lessened and the health and comfort of the population much benefited.

DR. CHARLES DAVIS HURT, one of Atlanta's representative physicians, died at his home in Inman Park, August 12th, after a lingering illness of eight months.

Dr. Hurt was graduated from the Medical College of Georgia in 1871. He practiced in Notasulga, Hurtsboro and Columbus, Ga., until 1892, when he located in Atlanta. In 1894 he was elected vice-president of the Atlanta Medical Society. For the past ten years he was a member of the staff of the Grady Hospital, and to him is due much of the credit for the establishment of the Wesley Memorial Hospital. Dr. Hurt was also a member of the Georgia Medical Association, and one of the faculty of the Atlanta School of Medicine. He was a Christian gentleman, a successful practitioner, and his loss will be keenly felt by his many friends and patients. We join them in extending to his family our deepest sympathy.

---

THE House, August 6th, passed the pure-food bill introduced by Mr. Wright of Floyd, and framed in accordance with the Act recently passed by the national congress. The measure was passed by a vote of 107 to 5, the whole of the morning session having been consumed in the consideration. This bill provides for the prevention of the adulteration, misbranding and imitation of foods for man or beast, of beverages, candies and condiments, of medicines, drugs and liquors, or their manufacture and sale in the State of Georgia. It prescribes penalties for the violations. It provides for the inspection and analysis of these various articles by the State Department of Agriculture. The Senate later adopted the bill with four amendments, the vote being without opposition.

